# Chirality of Novel Bitopic Agonists Determines Unique Pharmacology at the Dopamine D3 Receptor

**DOI:** 10.3390/biom11040570

**Published:** 2021-04-13

**Authors:** Pramisha Adhikari, Bing Xie, Ana Semeano, Alessandro Bonifazi, Francisco O. Battiti, Amy H. Newman, Hideaki Yano, Lei Shi

**Affiliations:** 1 Computational Chemistry and Molecular Biophysics Section, Molecular Targets and Medications Discovery Branch, National Institute on Drug Abuse—Intramural Research Program, National Institutes of Health, Baltimore, MD 21224, USA; pramisha.adhikari18@gmail.com (P.A.); bing.xie@nih.gov (B.X.); 2 Department of Pharmaceutical Sciences, School of Pharmacy, Bouvé College of Health Sciences, Northeastern University, Boston, MA 02115, USA; a.semeano@northeastern.edu; 3 Medicinal Chemistry Section, Molecular Targets and Medications Discovery Branch, National Institute on Drug Abuse—Intramural Research Program, National Institutes of Health, Baltimore, MD 21224, USA; alessandro.bonifazi2@nih.gov (A.B.); francisco.battiti@nih.gov (F.O.B.); anewman@intra.nida.nih.gov (A.H.N.)

**Keywords:** dopamine D3 receptor, dopamine D2 receptor, bitopic ligand, biased agonism, functional selectivity, subtype selectivity, subtype affinity, chirality

## Abstract

The dopamine D2/D3 receptor (D_2_R/D_3_R) agonists are used as therapeutics for Parkinson’s disease (PD) and other motor disorders. Selective targeting of D_3_R over D_2_R is attractive because of D_3_R’s restricted tissue distribution with potentially fewer side-effects and its putative neuroprotective effect. However, the high sequence homology between the D_2_R and D_3_R poses a challenge in the development of D_3_R selective agonists. To address the ligand selectivity, bitopic ligands were designed and synthesized previously based on a potent D_3_R-preferential agonist PF592,379 as the primary pharmacophore (PP). This PP was attached to various secondary pharmacophores (SPs) using chemically different linkers. Here, we characterize some of these novel bitopic ligands at both D_3_R and D_2_R using BRET-based functional assays. The bitopic ligands showed varying differences in potencies and efficacies. In addition, the chirality of the PP was key to conferring improved D_3_R potency, selectivity, and G protein signaling bias. In particular, compound AB04-88 exhibited significant D_3_R over D_2_R selectivity, and G protein bias at D_3_R. This bias was consistently observed at various time-points ranging from 8 to 46 min. Together, the structure-activity relationships derived from these functional studies reveal unique pharmacology at D_3_R and support further evaluation of functionally biased D_3_R agonists for their therapeutic potential.

## 1. Introduction

Dopamine (DA) is a major neurotransmitter in the central nervous system responsible for various physiological functions such as motor control, cognition, reward, pain, and memory and learning [1]. Dopamine signaling is mediated by five G protein-coupled receptors (GPCRs) classified into two subgroups based on distinct sequence homologies and signal transduction activities [2]: D_1_-like receptors that primarily couple to Gs protein and enhance the activity of adenylyl cyclase, leading to an increase in intracellular cAMP production, and D_2_-like receptors that primarily couple to the Gi/Go class of G proteins and suppress the activity of adenylyl cyclase. The dopamine D_2_ and D_3_ receptors (D_2_R and D_3_R), both belonging to the D_2_-like receptor subfamily, represent the major targets for neuropsychiatric disorders such as schizophrenia, Parkinson’s disease (PD), and substance use disorders (SUDs) [3,4]. Between the two receptor subtypes, selective targeting of D_3_R would have lower potential for side effects because of its relatively restricted distribution in the ventral striatum compared to the expression of D_2_R both centrally and peripherally [5,6,7]. Additionally, D_3_R has also emerged as the target for L-DOPA induced dyskinesia (LID) in PD [8,9,10]. D_3_R is upregulated in animal models of PD, essentially in the same regions of the brain where D_1_R is expressed. As such, D_3_R-D_1_R heteromerization has been suggested to underlie the LID in PD [8,10].

While D_3_R antagonists or partial agonists have been studied for schizophrenia, substance use disorders, and L-DOPA induced dyskinesia in PD, D_3_R-preferential agonists, which is the focus of this study, have been studied for PD and other motor-associated disorders [3,11,12]. Particularly, D_3_R has emerged as a potential target for the treatment of PD due to their pharmacological similarity to the D_2_R, but without their added risk for the side-effects likely associated with the peripherally distributed D_2_Rs [5,6,13]. For example, D_3_R agonists have been shown to alleviate the symptoms of PD in a chemically induced mouse model of PD. Rescue of DA depletion in the striatum as well as DA neuronal death in the substantia nigra was posited as the mechanism of action of these agents. Importantly, these beneficial effects were not observed in the D_3_R knockout mice, thus validating the role of D_3_R in mediating these effects [14]. Thus, D_3_R agonists have been shown to not only improve PD symptoms, such as motor perturbation and cognitive deficits, but also appear to slow down the neurodegenerative process that underlies PD progression, in these animal models [13]. However, not all reports point to the beneficial roles of D_3_R, as the involvements of D_3_R in neuroinflammation and PD pathogenesis have been reported [4]. Despite tremendous efforts, the development of highly selective D_3_R agonists has remained a challenge, because of the nearly identical orthosteric binding site (OBS) and the 78% sequence identity in the transmembrane domain between the D_2_R and D_3_R [15,16,17]. This not only poses a challenge in discerning the individual contributions in conferring the therapeutic effects of D_2_R/D_3_R agonists, but also in understanding the receptor specific tissue distribution and signaling pathways such as dimerization, transactivation, biased signaling, and allosterism. Indeed, D_3_R signaling is very intricate that several ex vivo and in vitro studies demonstrate its existence as homomers and heteromers with either D_1_R or D_2_R [18,19,20]. The understanding of D_3_R specific signaling is further complicated by the distinct mechanisms of action and downstream functional profiles for homomers and heteromers [20,21]. These unique mechanisms of action may contribute to the therapeutic properties of antiparkinsonian agents as suggested by their high potency at D_2_R-D_3_R heteromers [22]. D_3_R agonists currently used as therapies or as research tools exhibit limited D_3_R selectivity of ~10 fold over D_2_R [2]. Thus, novel D_3_R agonists with high affinity, selectivity, over D_2_R and other homologous GPCRs (e.g., 5HT_1A_), are required to probe their unique pharmacological properties and to determine their therapeutic efficacy as well as side effect profiles.

One important concept in GPCR pharmacology is functional selectivity, whereby GPCR biased ligands selectively modulate canonical G protein dependent pathways or G protein independent pathways such as β-arrestin signaling [23,24]. Increasing evidence suggests that functional selectivity may provide increased efficacy, improved safety profiles, and overall therapeutic advantage [25]. Indeed, G protein biased agonism at D_2_R has been suggested to underlie antipsychotic efficacy [26,27,28]. At D_3_R, a series of agonists have been synthesized and identified such as SK609 and SK213 with higher potency for G protein dependent pathways and low tendency to recruit β-arrestin. Among this series, studies using the lead D_3_R selective G protein biased agonist revealed efficacy in improving PD symptoms in hemiparkinsonian rodent models [29,30], indicating a potential therapeutic utility of G protein functional bias at D_3_R. In case of β-arrestin bias, there is no evidence suggesting therapeutic utility of D_3_R specific β-arrestin bias; however, studies in several other GPCRs indicate the potential therapeutic utility of β-arrestin bias in psychiatric and neurological disorders including schizophrenia, PD and SUDs [31].

Although several G protein versus β-arrestin biased agonists for D_3_R have been successfully identified using cellular functional assays, many studies lack a biophysical approach that can directly demonstrate coupling of G protein versus β-arrestin [29,32,33]. Thus, there is a need for biophysical characterization of novel agonists with D_3_R specific functional selectivity before evaluating whether such agonists can provide therapeutic value in in vivo models and beyond.

Bitopic ligands are comprised of a primary pharmacophore (PP) that binds to the OBS—the endogenous ligand binding site, and a secondary pharmacophore (SP) that binds to a secondary binding pocket (SBP), connected by a chemically defined linker [34,35,36]. Previous studies reveal that the bitopic ligand strategy can provide improved receptor subtype selectivity, affinity, and functional selectivity [37,38,39]. Additionally, bitopic ligands can also confer unique receptor signaling an example of which is the bitopic ligand SB269,652 that behaves as an allosteric antagonist at D_3_Rs and D_2_Rs [40,41]. Indeed, we have successfully utilized a bitopic design strategy to synthesize potent, selective, and G protein biased full agonists at D_2_R [42]. Using a similar strategy, we recently synthesized bitopic D_3_R compounds with increased binding affinity and selectivity for D_3_R demonstrated in radioligand binding studies [43]. In this study, we utilize bioluminescence resonance energy transfer (BRET) based biophysical functional assays to probe structure activity relationships (SAR) in conferring D_3_R over D_2_R functional selectivity.

**Figure 1:**
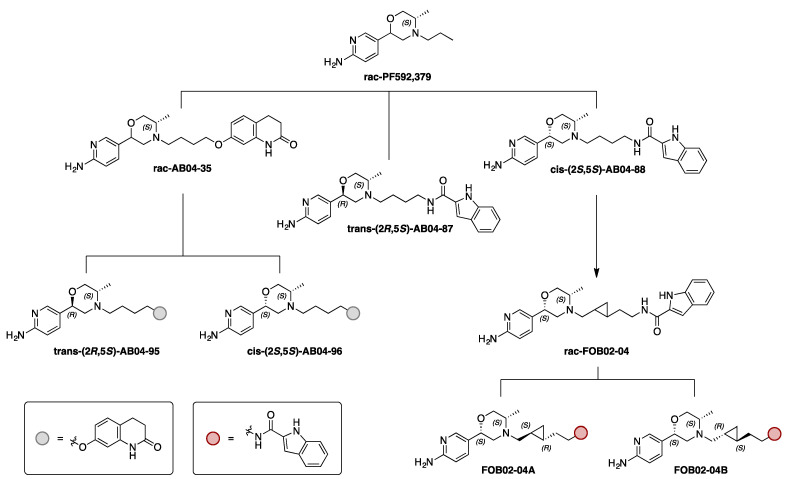
Drug design for D_3_R agonists. For the bitopic ligand design, the primary pharmacophore (PP) scaffold used was inspired by PF592,379. Several bitopic ligands based on the PF592,379 moiety were synthesized [43], among which the ligands shown were tested for functional characterization.

Specifically, we tested a series of congeneric bitopic compounds with their PP based on PF592,379 (Figure 1), an agonist at D_3_R developed by Pfizer for the treatment of sexual dysfunction and pain [43,44,45]. Among the series of compounds generated in Battiti et al. [43,46], we selected the ones that showed interesting SAR in radioligand binding assays for functional characterization [43]. In particular, we first selected the compounds that meets at least one of the two criteria: (i) a Ki < 32 nM in D_3_R binding (ii) a D_3_R over D_2_R selectivity > 22 fold. In order to comprehensively probe the role of chirality in conferring unique pharmacology, we further evaluated the corresponding racemic mixture or stereoisomers of the selected compounds in our BRET assays. First, a 3,4-dihydroquinoline-2(1*H*)-one SP, inspired by the antipsychotic D_2_R/D_3_R partial agonist Aripiprazole, was tethered to the PP with a butyl linker to generate rac-AB04-35, a mixture of two diastereoisomers. Based on the chirality at the PP morpholine ring, specifically in the 2-position, *trans*-(2*R*, 5*S*)-AB04-95 and *cis*-(2*S*, 5*S*)-AB04-96 were prepared via diastereospecific synthesis [43]. Second, a 2-indole amide SP was connected to the PP with the same butyl linker. In order to further assess the role of chirality in the PP, both *trans*-(2*R*, 5*S*)-AB04-87 and *cis*-(2*S*, 5*S*)-AB04-88 diastereoisomers were synthesized. Third, based on the observation of the optimal *cis*-(2*S*, 5*S*)-PP stereochemistry for D_3_R binding, the 2-indole amide SP was connected via a more rigid *trans*-cyclopropyl [47] containing linker to generate rac-FOB02-04 as a diastereoisomeric mixture of the *trans* cyclopropyl ring. Chiral resolution of the cyclopropyl linker gave two different isomers (1*R*, 2*S*)-FOBO2-04A and (1*S*, 2*R*)-FOB02-04B (Figure 1). Investigating pairs of diastereoisomers, we report functionally selective bitopic compounds that only show the bias characteristic in one stereoisomer, underscoring the importance of stereochemistry as a fundamental structural characteristic in D_3_R functional selectivity.

## 2. Materials and Methods

### 2.1. Bioluminescence Resonance Energy Transfer (BRET) Studies

**Figure 2:**
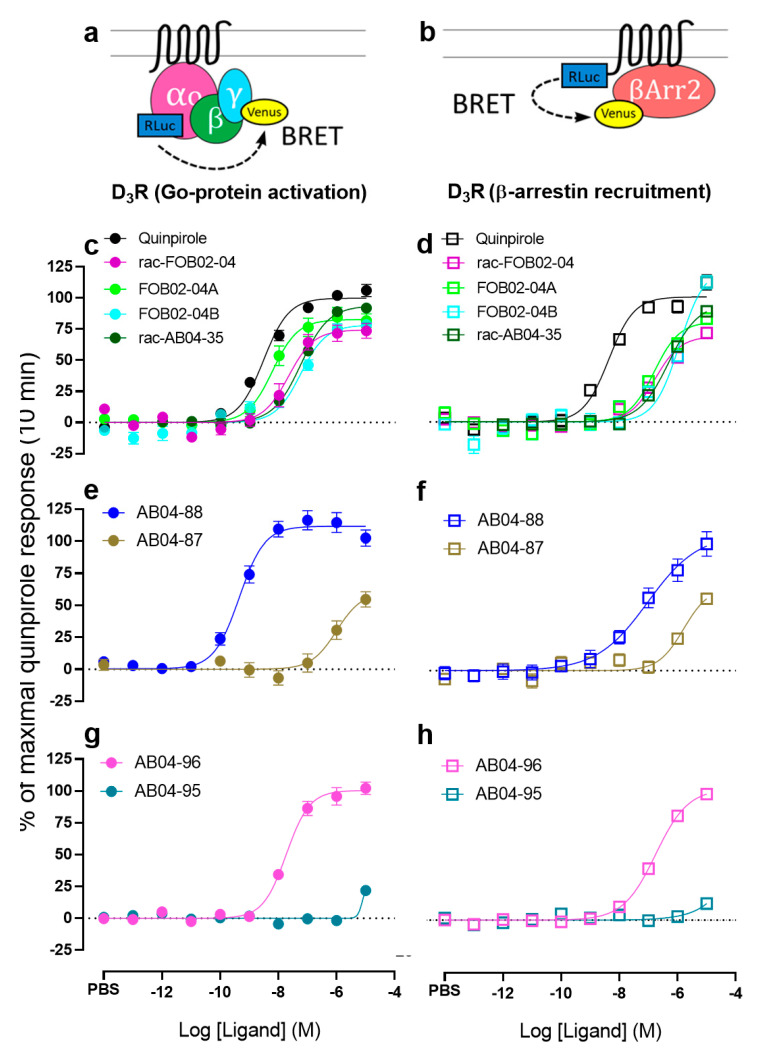
Pharmacological comparison of D_3_R bitopic ligands in both G protein activation and β-arrestin recruitment at 10 min. (**a**). Scheme for the bioluminescence resonance energy transfer (BRET) between Gαo-RLuc and Gγ-Venus. (**b**). Scheme for the BRET between D_3_R-RLuc and β-arrestin2-Venus. Concentration-response curves (CRCs) of drug-induced BRET at 10 min between Gαo-RLuc and Gγ-Venus (**c**,**e**,**g**). CRCs of drug-induced BRET at 10 min between D_3_R-RLuc and β-arrestin2-Venus (**d**,**f**,**h**). CRCs are plotted as percentage of maximal response by quinpirole and presented as means ± SEM of *n* ≥ 3 independent experiments.

The BRET-based Go protein activation and β-arrestin recruitment assays were performed as described previously [34,42]. Go protein activation assay uses Renilla luciferase 8 (Rluc8; provided by Dr. S. Gambhir, Stanford University, Stanford, CA, USA)-fused Gα_oA_ and mVenus-fused Gγ_2_ as the BRET pair (Figure 2a). β-arrestin recruitment assay uses RLuc8-fused D_3_R or D_2_R and mVenus-fused β-arrestin2 as the BRET pair (Figure 2b). HEK293T cells were transiently transfected with the above constructs using polyethyleneimine (PEI) at a ratio of 2:1 (PEI:total DNA by weight). After ~48 h of transfection, cells were washed, harvested, and resuspended in PBS + 0.1% glucose + 200 µM Na bisulfite buffer. Then, 200,000 cells were transferred to each well of the 96-well plates (White Lumitrac 200, Greiner bio-one, Monroe, NC, USA) followed by addition of 5 µM coelenterazine H, a luciferase substrate for BRET. Test compounds, reference D_2_/D_3_ agonist-quinpirole (Tocris Bioscience, Minneapolis, MN, USA), and vehicle controls were then added by Nimbus liquid handling system (Hamilton, Reno, NV, USA) with its stamping protocol and cells were incubated at 37 °C for 10 min. BRET signal was then measured using a Pherastar *FSX* plate reader (BMG Labtech, Cary, NC, USA). For kinetic experiments, cells were incubated at 37 °C within the Pherastar *FSX* plate reader (BMG Labtech, Cary, NC, USA) with BRET signal measurements taken at various time-points ranging from 2–46 min. BRET ratio was calculated as the ratio of mVenus (530 nm) over RLuc8 (480 nm) emission.

Data were collected from at least 3 independent experiments and normalized to maximal response by quinpirole as 100% and response by vehicle as 0%. Concentration response curves (CRCs) were generated using a non-linear sigmoidal dose-response analyses using Prism 8 (GraphPad Software, San Diego, CA, USA) and presented as mean ± SEM.

### 2.2. Bias Factor Analysis

To evaluate whether the test compounds exhibited G protein versus β-arrestin signaling bias, bias factors were calculated using the method as follows [1]:

(1) $$biased\;activity=\;log_{10}{\left(\frac{E_{max}}{EC_{50}}\right)}_{G\;protein}-log_{10}{\left(\frac{E_{max}}{EC_{50}}\right)}_{\beta \;arrestin}$$

An arbitrary but stringent cut-off of ≥ ±2.0 (in logarithmic scale) was chosen to identify biased ligands. Bias factor values >2.0 represent bias towards G protein while values below <−2.0 represent bias towards β-arrestin.

### 2.3. An In-House Program for Kinetics Analysis of Functional Assay Data

In this study, the BRET signals for each 96-well plate were detected every two minutes using the BMG Pherastar *FSX* plate reader (BMG Labtech, Cary, NC, USA), resulting in 23 datasets for a 46 min measurement in one raw data file per plate. Such amount of data is beyond what can be conveniently and reliably processed by manual extraction, transformation, and normalization, before the regression analysis by Prism. Thus, we developed an in-house python program that can process and analyze the kinetics of functional assay data. While this program was configurated to fit the raw file output format of the plate reader used in this study, it can be easily adapted to process other output formats, time intervals, and plate maps (i.e., how the rows and columns of 96-well plates are configured for the dose-response measurements). This program also has the capability to process multiple files, based on predefined file locations in a configuration file. In this configuration file, for each raw file to be processed, it also includes time intervals, receptor construct, test compounds, and concentration ranges for each plate.

Each raw BRET data file was first preprocessed by detecting the data set for each time cycle. The response values were calculated as the ratio between 475-30B and 535-30A data for each well of the 96-well plate in the same time cycle. We took the average of response values for each compound at each concentration in the same time cycle. We then fitted response values to the sigmoidal dose-response function.

Sigmoidal dose-response function: $S\left(x\right)=\frac{top-bottom}{1+10^{\left(x-logEC50\right)}}+bottom$, top and bottom are the maximum and minimum of the response values, respectively, x is the logarithm of the concentration, and logEC_50_ is the x value when the response is halfway between bottom and top.

We used the scipy.optimize.curve_fit module (version 1.5.2) [48] to perform this fitting process. In this curve fit module, we chose ‘lm’ as the optimization method type, which can replicate the regression result using Prism 8 (GraphPad Software, San Diego, CA, USA). The initial guess values were the minimum response value for “bottom”, the maximum response value for “top”, and the halfway value of the log(concentration) range for “logEC_50_”. After the fitting, the *E*_max_ was calculated as the difference between the optimized “bottom” and the optimized “top”, and the log (concentration) resulting in half of *E*_max_ is the logEC_50_.

To demonstrate functional kinetics, the program integrates the regression results at each time point and plots the *E*_max_ and logEC_50_ evolutions along the time (Figure 4).

### 2.4. Statistics

Statistical significance values were calculated using Prism 8 (GraphPad Software, San Diego, CA, USA)’s ordinary One-way ANOVA (independent variable: compound treatment, dependent variables: efficacy or pEC_50_s) followed by Dunnett’s multiple comparisons tests. For kinetic data, statistical significance values were calculated using GraphPad Prism’s ordinary two-way ANOVA (independent variables: compound treatment and time-point, dependent variables: efficacy or pEC_50_s) followed by Sidak’s multiple comparisons tests. For the above, two multiple comparisons, ‘*’ represents a significance of *p* < 0.05; ‘**’ of *p* < 0.01; ‘***’ of *p* < 0.001 and ‘****’ of *p* < 0.0001 compared to quinpirole. Dunnett’s multiple comparisons tests were also performed against AB04-87 with ‘δ’ representing significance of *p* < 0.05; ‘δδ’ of *p* < 0.01; ‘δδδ’ of *p* < 0.001 and ‘δδδδ’ of *p* < 0.0001 compared to AB04-87. Data are reported from more than three experiments performed in triplicate. In the case where the data points could not be fitted into the non-linear sigmoidal dose-response equation, the pharmacological parameters are reported as ND (not determined).

## 3. Results

### 3.1. Bitopic Compounds Exhibit Varying Pharmacological Profiles at Both D_3_R and D_2_R Compared to the Reference D_2_R/D_3_R Agonist Quinpirole

The bitopic compounds characterized in this study all have the PF592,379 scaffold as the primary pharmacophore (PP). For the chiral center at the 2-position of the morpholine ring in this scaffold, while AB04-88, rac-FOB02-04, FOB02-04A, FOB02-04B, and AB04-96 are in the *cis* conformation, AB04-95 and AB04-87 possess *trans* stereochemistry. rac-AB04-35 is the diastereomeric mixture of the AB04-95 and AB04-96, which have the same butyl linker and 3,4-dihydroquinoline-2(1*H*)-one as the SP (Figure 1). While AB04-87 and AB04-88 also have the same butyl linker, they differ from these compounds in their SP (2-indole amide). rac-FOB02-04, FOB02-04A, and FOB02-04B have the same 2-indole amide SP but with different *trans*-cyclopropyl containing linker. To investigate the impact of these differences in stereochemistry, linker, and SP on their functional properties, we evaluated the pharmacological profiles of these bitopic compounds with BRET-based Go protein activation and β-arrestin recruitment assays (see Section 2).

In the D_3_R Go protein activation assay, our results showed that except for AB04-88, rac-AB04-35, and AB04-96, the others exhibited lower efficacies than that of quinpirole (*p* < 0.01 in all cases, one-way ANOVA followed by Dunnett’s multiple comparisons test, Figure 2c,e,g, Table 1). While rac-FOB02-04, FOB02-04B, AB04-87, rac-AB04-35, and AB04-96 exhibited statistically lower potencies compared to that of quinpirole (*p* < 0.001 in all cases), FOB02-04A showed comparable potency to that of quinpirole. Interestingly, AB04-88 exhibited a significantly higher potency (6.5-fold) than that of quinpirole (*p* < 0.001, Figure 2e, Table 1).

In D_3_R β-arrestin recruitment assay, rac-FOB02-04, FOB02-04A, and AB04-87 showed significantly lower efficacies compared to quinpirole (*p* < 0.05 in all cases, Figure 2d,f, Table 1). FOB02-04B, AB04-88, rac-AB04-35, and AB04-96 showed comparable efficacies compared to quinpirole. All bitopic ligands showed significantly lower potencies in the β-arrestin recruitment assay compared to that of quinpirole (*p* < 0.0001,Figure 2d,f,h, Table 1). AB04-95 showed the lowest efficacy profile at both D_3_R mediated Go protein activation as well as β-arrestin recruitment.

In the D_2_R Go protein activation assay, FOB02-04B and rac-AB04-35 exhibited statistically lower efficacies as well as potencies compared to quinpirole (*p* < 0.001 in both cases, One-way ANOVA followed by Dunnett’s multiple comparisons test, Table 2). rac-FOB02-04 showed a statistically lower potency (*p* < 0.05) with comparable efficacy compared to quinpirole. Both the bitopic ligands with the *trans* diastereoisomer in the PP: AB04-87 and AB04-95 did not show any activity in both Go protein activation and β-arrestin recruitment. All the remaining bitopic ligands showed significantly lower potencies compared to quinpirole (*p* < 0.0001 in all cases) in β-arrestin recruitment (Table 2). While rac-FOB02-04, FOB02-04A, FOB02-04B and AB04-88 showed lower efficacies (*p* < 0.0001), rac-AB04-35 showed comparable efficacy to quinpirole in β-arrestin recruitment. Interestingly, AB04-96 exhibited a ~22% higher efficacy compared to quinpirole (*p* < 0.001) in D_2_R mediated β-arrestin recruitment. Of note, this is the only compound that exhibits higher efficacy than quinpirole.

The *cis*-isomers of bitopic ligands are more potent at D_3_R than their *trans*-isomers.

AB04-96 and AB04-95 only differ in the chirality at the 2-position of the morpholine ring in their PP, i.e., in *cis-* and *trans*-stereochemistry, respectively (Figure 1) [43]. The same difference in stereochemistry is seen between AB04-88 and AB04-87, another diastereoisomeric pair. In all four compounds, the same butyl linker was used to connect the PP with their corresponding SPs. Given that chirality can considerably modulate pharmacological profiles [38,49], we compared these two pairs of ligands in their ability to promote D_3_R Go protein activation and β-arrestin recruitment.

As shown in Figure 2e–h and Table 1, in both cases, the *cis*-stereochemistry (AB04-88 and AB04-96) of the morpholine ring confer higher potencies as well as efficacies compared to their corresponding *trans* diastereoisomers (AB04-87 and AB04-95, respectively). The *cis*-AB04-88 exhibited a ~2188-fold increase in potency (*p* < 0.0001, Dunnett’s multiple comparisons test) and an increase in *E*_max_ of ~51.3% (*p* < 0.0001) compared to its *trans*-AB04-87 in D_3_R Go protein activation (using the efficacy of quinpirole as 100%) (Figure 2e and Table 1).

In D_3_R mediated β-arrestin recruitment, the *cis*-AB04-88 exhibited a significant increase in *E*_max_ of ~42.8% (*p* < 0.0001) and a 17-fold increase in potency compared to its *trans*-AB04-87 (Figure 2f and Table 1). Interestingly, while the *trans*-AB04-95 exhibited a near complete abolishment of D_3_R mediated Go protein activation as well as β-arrestin recruitment, *cis*-AB04-96 exhibited a robust activity in both assays as shown in Figure 2g–h and Table 1. Thus, the potency and efficacy of AB04-95 cannot be quantitatively determined.

### 3.2. The cis-AB04-88 but not the trans-AB04-87 Shows an Improved D_3_R Selectivity Over D_2_R

**Figure 3:**
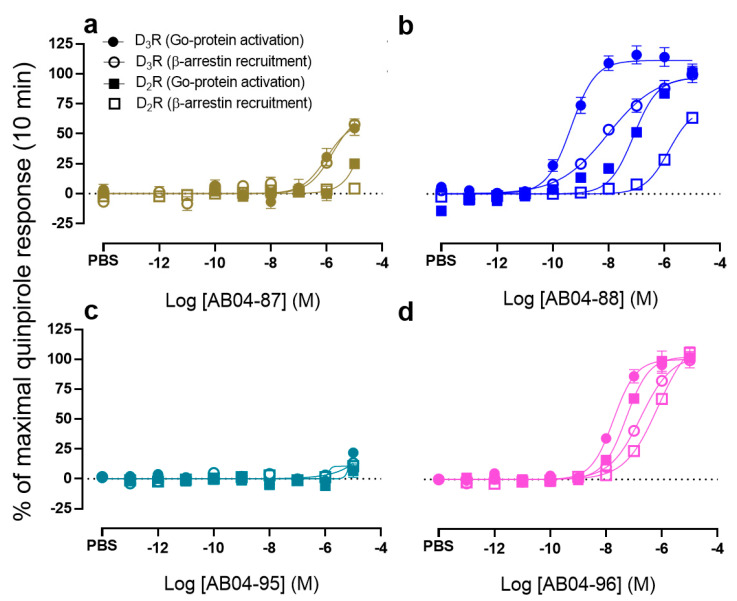
Pharmacological comparison of *cis* and *trans* pairs in both D_3_R and D_2_R mediated G protein activation and β-arrestin recruitment at 10 min. CRCs of AB04-87 (**a**), AB04-88 (**b**), AB04-95 (**c**) and AB04-96 (**d**)-induced BRET at 10 min between Gαo-RLuc and Gγ-Venus and D_3_R or D_2_R-Rluc and β-arrestin2-Venus. CRCs are plotted as percentage of maximal response by quinpirole and presented as means ± SEM of *n* ≥ 3 independent experiments.

SPs and linkers are key to achieving subtype selectivity by binding to the SBPs unique to each receptor subtype [38,50,51,52]. To investigate whether these ligands exhibit D_3_R over D_2_R subtype selectivity, their ability to promote D_3_R and D_2_R mediated Go protein activation and β-arrestin recruitment were compared. In Go protein activation, all the compounds except AB04-88 and quinpirole showed < 10-fold D_3_R over D_2_R selectivity (Table 3). While the *cis*-stereochemistry of the PP, i.e., AB04-88 exhibited ~123-fold D_3_R over D_2_R selectivity in Go protein activation, selectivity with the *trans*-isomer could not be quantified (Figure 3a–b and Table 3) due to loss of Go protein activation at D_2_R (Table 2).

AB04-96 is the bitopic ligand with the same *cis*-stereochemistry of PP and butyl linker as AB04-88 but with a different SP. Interestingly, this *cis*-isomer only exhibited a 2.8-fold selectivity (Figure 3d and Table 3) with its corresponding *trans*-isomer showing near abolishment of activity at both D_3_R and D_2_R (Figure 3c and Table 1 and Table 2).

In β-arrestin recruitment, quinpirole, rac-FOB02-04, and AB04-88 exhibited > 10-fold D_3_R over D_2_R selectivity. Conversely, bitopic ligands FOB02-04A, FOB02-04B, rac-AB04-35, and AB04-96 exhibited < 10-fold selectivity. D_3_R over D_2_R selectivities in β-arrestin recruitment could not be determined for AB04-87 and AB04-96 because of their near abolishment of activity at either D_2_R only (AB04-87) or at both D_3_R and D_2_R (AB04-96) (Figure 3a,c and Table 1, Table 2 and Table 3).

### 3.3. The cis-AB04-88 But Not the trans-AB04-87 Shows D_3_R Selective Go Protein Bias

Previous studies have shown that bitopic ligands can not only improve subtype potency and selectivity but can also promote functional selectivity [42,52]. There is an increasing interest in understanding G protein versus β-arrestin biased agonism at GPCRs because of potential therapeutic utility upon activating one pathway over the other. To evaluate whether the compounds shown in Figure 1 can promote D_3_R specific G protein versus β-arrestin functional selectivity, bias factors were derived using the model that incorporates both efficacy and potency differences into a single index [1].

The biased activity equation used to evaluate G protein versus β-arrestin bias is described in the Section 2. An arbitrary but stringent cut-off of > ± 2.0 (in logarithmic scale) was chosen to identify biased ligands, where values > 2.0 represent bias towards G protein while values < −2.0 represent bias towards β-arrestin. The calculated biased factors are presented in Table 3.

Among all the ligands tested for bias at both D_3_R and D_2_R (Table 3), only the *cis*-isomer AB04-88 exhibited a biased factor equal to 2.3 (Figure 3b and Table 3) at D_3_R, suggesting that this ligand promotes D_3_R mediated Go protein bias. Upon inversion at the chiral center in the 2-position to generate *trans*-isomer AB04-87, the D_3_R mediated Go protein bias is completely lost (Figure 3a and Table 3). A similar trend can be observed at D_2_R, whereby the bias factor for AB04-88 was calculated to be 1.5 whereas the bias factor for AB04-87 could not be quantified (Figure 3a–b and Table 3), suggesting that AB04-88 exhibits a D_3_R selective Go protein bias.

In case of the second set of *cis*-*trans* pairs, the bias factor for the *cis-*AB04-96 was calculated to be 1.0 and 1.1 for D_3_R and D_2_R, respectively. However, the bias factors for its corresponding *trans*-isomer AB04-95 could not be quantified at both D_3_R and D_2_R because its activities were nearly abolished for both Go protein activation and β-arrestin recruitment. Although AB04-88 and AB04-96 were designed using the same *cis* form of the PP and the same butyl linker, they differ in their SPs. The observed D_3_R mediated Go protein bias using AB04-88 but not AB04-96 further stresses the role of SPs in conferring unique pharmacological profiles.

### 3.4. Time-Dependent Pharmacological Analysis Reveals Higher Go Protein Activation E_max_ for AB04-88 at Later Time Points

Time-dependent pharmacological profile, often called kinetic context, can be determined by the ligand-binding and receptor-signaling kinetics collectively and can impact biased agonism and its quantification [53,54]. Since our bitopic agonist AB04-88 exhibited D_3_R specific bias towards Go protein, we evaluated the kinetic profiles of AB04-88 in comparison to that of quinpirole. We assessed the kinetic profiles of both efficacies and potencies that constitute the two components used in our D_3_R biased agonism quantification [1], based on the data points collected every 2 min up to 46 min (see Section 2).

**Figure 4:**
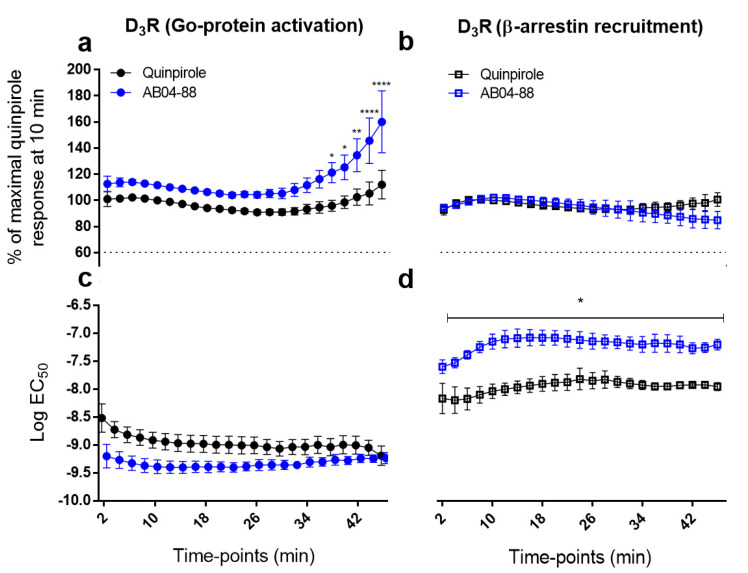
Time-dependent pharmacological profiles of AB04-88 and quinpirole on D_3_R mediated G protein activation and β-arrestin recruitment. a-c. Efficacy (**a**,**b**) and potency (**c**,**d**) profiles of D_3_R mediated G protein activation (**a**,**c**) and β-arrestin recruitment (**b**,**d**) measured with 2 min interval from 2 to 46 min. The *E*max values are plotted as percentage of maximal response by quinpirole at 10 min. Plots are presented as means ± SEM of *n* ≥ 3 independent experiments. Statistical significances were calculated using GraphPad Prism’s ordinary two-way ANOVA (in each case, **** *p* < 0.0001) followed by Sidak’s multiple comparisons tests with ‘*’ representing significance of *p* < 0.05; ‘**’ of *p* < 0.01 and ‘****’ of *p* < 0.0001 compared to that of quinpirole.

As shown in Figure 4, there were no significant differences between the AB04-88 and quinpirole efficacies at D_3_R until the 36 min time-point. Interestingly, after the 36 min time-point, AB04-88 exhibits significantly higher efficacies compared to quinpirole (in all cases *p* < 0.05; two-way ANOVA followed by Sidak’s multiple comparisons test) with maximal increases of > 40% observed at 44 and 46-min time-points (Figure 4a and Table 4). In contrast, there were no statistically significant differences observed between AB04-88 and quinpirole for EC_50_ of Go protein activation (Figure 4c and Table 4) or the efficacy component for β-arrestin recruitment (Figure 4b and Table 4). In contrast, in the β-arrestin recruitment assay, the *E*_max_ profiles for both AB04-88 and quinpirole nearly overlap up to 36 min, with AB04-88 showing slightly decreased *E*max afterwards. The potency of AB04-88 remains significantly and consistently lower compared to quinpirole at all time-points ranging from 4–46 min (in all cases *p* < 0.05; two-way ANOVA followed by Sidak’s multiple comparisons test) (Figure 4d and Table 4). 

We also calculated the bias factors at all time-points, AB04-88 exhibited a bias at timepoints ranging from 8–46 min (> ±2.0) but without major changes.

These data collectively suggest that, in the case of AB04-88, kinetics analysis revealed a significant *E*_max_ increase for Go protein activation at later time points, which, however, did not significantly affect the bias factor.

## 4. Discussion

The discovery of D_3_R selective agonists is of particular interest because such compounds would help ascertain D_3_R specific physiological and pathological functions from that of D_2_R in neurodegenerative disorders such as PD [55]. Furthermore, such agonists may exhibit improved clinical utility in the treatment of such disorders. The bitopic D_3_R ligands evaluated in this study exhibit varying pharmacological profiles compared to the reference D_2_R/D_3_R full agonist quinpirole. The PPs of the bitopic ligands in this study are based on the D_3_R-preferential agonist, PF592,379 and are either a mixture of diastereoisomers or one of the two *cis*- and *trans*- isomers based on the chirality of its morpholine ring. In addition, the bitopic ligands differ in their SPs, linkers, and linker stereochemistry.

Comparing the diastereomer pairs within the same ligand structures, the (2*S*, 5*S*) enantiomer of the PP morpholine ring shows more efficacious and potent D_3_R G protein signaling. This observation confirms the significance of enantiospecificity in the morpholine ring in achieving D_3_R activation efficacy.

In D_3_R Go protein activation BRET, *cis*-(2*S*, 5*S*)-AB04-88 presenting a 2-indole amide SP connected by a butyl linker, showed a 123-fold selectivity for D_3_R over D_2_R. Interestingly, another compound AB04-96 that has the same PP and linker but with a 3,4-dihydroquinolin-2(1*H*)-one SP only shows a 2.8-fold D_3_R over D_2_R selectivity. This observation highlights the importance of the SP in promoting subtype selectivity where the 2-indole amide, but not 3,4-dihydroquinolin-2(1*H*)-one promotes D_3_R subtype selectivity. This is consistent with previous findings where the presence of a 3,4-dihydroquinolin-2(1*H*)-one moiety improves D_2_R subtype binding [42,56,57]. Unlike the previous radioligand binding study in which FOB02-04A showed a 47.5-fold D_3_R over D_2_R selectivity, we only observed an eight-fold selectivity in Go protein activation BRET. The binding affinities and selectivities observed in radioligand binding studies may not always correlate with cell-based functional assays. The observed discrepancies in these two types of assays may be explained by inherent differences of probe dependency and specific assay conditions [58].

Given the increasing evidence supporting therapeutic utility of functional selectivity in GPCRs, we evaluated the D_3_R bitopic agonists for their signaling bias. AB04-88, which showed the highest potency in D_3_R Go protein activation among all bitopic ligands studied, also exhibited a Go protein activation over β-arrestin recruitment bias by two log units. While the (2*S*, 5*S*) *cis* stereoisomer of the PP appears favorable for Go protein activation over β-arrestin recruitment, AB04-96 with the same *cis* conformation of PP and linker, but a different SP, does not exhibit functional bias. Similar to the D_3_R over D_2_R selectivity, this difference highlights the significance of the SP in conferring functional bias as well. Future computational studies with the two diastereoisomeric (based on PP morpholine ring) sets of D_3_R bitopic agonists will provide clues as to the interplay between chirality and SP chemistry to provide unique pharmacology.

Increasing evidence suggests the influence of ligand binding kinetics and receptor signaling in biased agonism [53,54]. Since AB04-88 exhibited D_3_R specific Go protein bias at 10 min, we investigated whether this bias would change in a time-dependent manner. The only kinetic profile affected was that of the efficacy of AB04-88 in D_3_R Go protein activation, where it significantly increased compared to quinpirole after 36 min. However, the bias factor was not affected due to the largely stable potencies in both Go protein activation and β-arrestin assays, the difference of which would be the dominant component in the factor when AB04-88 remained a strong partial agonist at the later time-points of β-arrestin recruitment.

Together, our newly developed D_3_R bitopic ligands provide novel tools to further probe the unique pharmacology, specific cellular signaling, and therapeutic potential of D_3_R.

## Figures and Tables

**Table 1:** Pharmacological comparison of bitopic ligands at D3R.

D_3_R, 10 min	Go Protein Activation Assay				β-arrestin Recruitment			
Compounds	*E*_max_ ± SEM (% of Quinpirole)	pEC_50_ ± SEM	Change *E*_max_ over Quinpirole	Fold Potency over Quinpirole	*E*_max_ ± SEM (% of Quinpirole)	pEC_50_ ± SEM	Change *E*_max_ over Quinpirole	Fold Potency over Quinpirole
**Quinpirole**	100 ± 2.27 ^δδδδ^	8.53 ± 0.08 ^δδδδ^	0	1.000	100 ± 2.6 ^δδδδ^	8.36 ± 0.09 ^δδδδ^	0	1.000
**rac-FOB02-04**	74.4 ± 3.4 ***	7.67 ± 0.16 ***,^δδδδ^	−25.6	0.138	68.5 ± 1.8 ***	6.78 ± 0.07 ****, ^δδδ^	−31.5	0.026
**FOB02-04A**	82.8 ± 3.0 **^,δδ^	8.22 ± 0.12 ^δδδδ^	−17.2	0.490	80.2 ± 3.3 *	6.80 ± 0.10 ****, ^δδδδ^	−19.8	0.028
**FOB02-04B**	78.4 ± 3.2 ****^,δδ^	7.25 ± 0.11 ****, ^δδδδ^	−21.6	0.052	112 ± 7.0 ^δδδδ^	5.87 ± 0.10 ****	33.2	0.003
**AB04-87**	60.3 ± 8.1 ****	6.00 ± 0.23 ****	−39.7	0.003	67.2 ± 8.2 ****	5.80 ± 0.19 ****	−32.8	0.003
**AB04-88**	111.6 ± 2.8 ^δδδδ^	9.34 ± 0.09 ***,^δδδδ^	11.6	6.500	110 ± 0.9 ^δδδδ^	7.03 ± 0.10 ****, ^δδδδ^	10.0	0.047
**rac-AB04-35**	93.9 ± 3.0 ^δδδδ^	7.24 ± 0.07 ****, ^δδδδ^	−6.1	0.051	94.8 ± 3.7 ^δδ^	6.31 ± 0.07 ****, ^δ^	−5.2	0.009
**AB04-95**	ND	ND	ND	ND	ND	ND	ND	ND
**AB04-96**	100.4 ± 2.7 ^δδδδ^	7.73 ± 0.08 ***, ^δδδδ^	0.4	0.158	104.0 ± 2.9 ^δδδ^	6.76 ± 0.06 ****, ^δδδ^	4.0	0.025

Mean *E*_max_ ± SEM and pEC50 ± SEM values along with fold changes over the reference D2R and D3R agonist—quinpirole are reported. Using Dunnett’s multiple comparisons tests, statistical significance are reported as ‘*’ representing significance of *p* < 0.05; ‘**’ of *p* < 0.01; ‘***’ of *p* < 0.001 and ‘****’ of *p* < 0.0001 compared to quinpirole, and ‘^δ^’ of *p* < 0.05; ‘^δδ^’ of *p* < 0.01; ‘^δδδ^’ of *p* < 0.001 and ‘^δδδδ^’ of *p* < 0.0001 compared to AB04-87. ND, not determined.

**Table 2:** Pharmacological comparison of bitopic ligands at D2R.

D_2_R, 10 min	Go Protein Activation Assay				β-arrestin Recruitment			
Compounds	*E*_max_ ± SEM (% of Quinpirole)	pEC_50_ ± SEM	Change *E*_max_ over Quinpirole	Fold Potency over Quinpirole	*E*_max_ ± SEM (% of Quinpirole)	pEC_50_ ± SEM	Change *E*_max_ over Quinpirole	Fold Potency over Quinpirole
**Quinpirole**	100 ± 2.0	7.46 ± 0.07	0.00	1.00	100 ± 2.8	6.93 ± 0.08	0.0	1.00
**rac-FOB02-04**	93.2 ± 2.2	7.13 ± 0.06 *	−6.80	0.47	62.9 ± 1.9 ****	5.72 ± 0.07 ****	−37.1	0.06
**FOB02-04A**	91.2 ± 2.8	7.31 ± 0.08	−8.80	0.71	60.4 ± 1.9 ****	6.04 ± 0.07 ****	−39.6	0.13
**FOB02-04B**	81.4 ± 3.8 ***	6.44 ± 0.13 ****	−18.60	0.10	56.9 ± 1.4 ****	5.45 ± 0.05 ****	−43.1	0.03
**AB04-87**	ND	ND	ND	ND	ND	ND	ND	ND
**AB04-88**	95.2 ± 2.4	7.25 ± 0.07	−4.80	0.62	72.6 ± 2.6 ****	5.84 ± 0.06 ****	−27.4	0.08
**rac-AB04-35**	75.9 ± 5.3 ***	6.48 ± 0.13 ****	−24.10	0.10	102.8 ± 2.5	5.74 ± 0.06 ****	2.80	0.06
**AB04-95**	ND	ND	ND	ND	ND	ND	ND	ND
**AB04-96**	102.7 ± 4.0	7.28 ± 0.11	2.70	0.66	121.6 ± 8.2 ***	6.14 ± 0.13 ****	21.6	0.16

Mean *E*_max_ ± SEM and pEC50 ± SEM values along with fold changes over quinpirole are reported. Using Dunnett’s multiple comparisons tests, statistical significance are reported as ‘*’ representing significance of *p* < 0.05; ‘***’ of *p* < 0.001 and ‘****’ of *p* < 0.0001 compared to quinpirole. ND, not determined.

**Table 3:** D3R over D2R selectivity and Go protein vs. β-arrestin bias factors of bitopic ligands.

	Selectivity (D_2_R/D_3_R)		Bias Factors	
Compounds	Go Protein Activation	β-arrestin Recruitment	D_3_R	D_2_R
**Quinpirole**	11.7	26.9	ND	ND
**rac-FOB02-04**	3.5	11.5	0.9	1.6
**FOB02-04A**	8.1	5.8	1.4	1.4
**FOB02-04B**	6.5	2.6	1.1	1.1
**AB04-87**	ND	ND	0.2	ND
**AB04-88**	123.0	15.5	2.3	1.5
**rac-AB04-35**	5.8	3.7	0.9	0.6
**AB04-95**	ND	ND	ND	ND
**AB04-96**	2.8	4.2	1.0	1.1

D3R over D2R selectivity and Go protein versus β-arrestin bias factors are reported. The value > 100 for D3R over D2R selectivity is highlighted in green. Bias factors were calculated as described in Section 2. Bias factor value > 2.0 is highlighted in orange. ND = not determined.

**Table 4:** Time dependent D3R pharmacological profile of quinpirole and AB04-88.

	Quinpirole					AB04-88				
	Go Protein Activation		β-arrestin Recruitment		Bias Factors	Go Protein Activation		β-arrestin Recruitment		Bias Factors
Time-points (min)	pEC_50_ ± SEM	*E*_max_ (% of quinpirole)	pEC_50_ ± SEM	*E*_max_ (% of quinpirole)		pEC_50_ ± SEM	*E*_max_ (% of quinpirole)	pEC_50_ ± SEM	*E*_max_ (% of quinpirole)	
**2**	8.51 ± 0.25	101 ± 5.6	8.20 ± 0.26	92.8 ± 3.9	0.4	9.20 ± 0.21	112.7 ± 5.8	7.64 ± 0.12	94.4 ± 1.4	1.6
**4**	8.72 ± 0.14	101.4 ± 2.0	8.23 ± 0.23	97.9 ± 1.9	0.5	9.27 ± 0.14	113.8 ± 3.3	7.57 ± 0.08 *	96.4 ± 0.2	1.8
**6**	8.81 ± 0.13	102.3 ± 1.0	8.21 ± 0.19	100.3 ± 1	0.6	9.32 ± 0.12	114.0 ± 1.6	7.43 ± 0.07 **	99.1 ± 0.2	1.9
**8**	8.86 ± 0.13	101.4 ± 0.4	8.13 ± 0.14	100.6 ± 0.3	0.7	9.37 ± 0.12	112.9 ± 0.6	7.30 ± 0.09 ***	101.3 ± 0.1	2.1
**10**	8.91 ± 0.14	100.0 ± 0.0	8.07 ± 0.12	100.0 ± 0	0.8	9.39 ± 0.12	111.7 ± 0.0	7.20 ± 0.13 ***	102.2 ± 0	2.3
**12**	8.94 ± 0.14	98.9 ± 0.3	8.04 ± 0.10	99.4 ± 0.3	0.9	9.40 ± 0.11	110.0 ± 0.5	7.16 ± 0.15 ***	101.8 ± 0.9	2.3
**14**	8.96 ± 0.15	97.3 ± 0.6	8.01 ± 0.11	98.3 ± 0.6	0.9	9.40 ± 0.10	108.9 ± 0.8	7.14 ± 0.16 ***	100.7 ± 1.8	2.3
**16**	8.97 ± 0.15	95.6 ± 0.9	7.97 ± 0.11	97.0 ± 0.9	1.0	9.39 ± 0.10	107.6 ± 1.2	7.13 ± 0.14 ***	100.2 ± 2.8	2.3
**18**	8.98 ± 0.15	94.2 ± 1.1	7.94 ± 0.12	95.9 ± 1.3	1.0	9.39 ± 0.10	106.4 ± 1.5	7.13 ± 0.13 ***	99.1 ± 3.3	2.3
**20**	8.99 ± 0.14	93.5 ± 1.2	7.92 ± 0.14	95.5 ± 1.5	1.0	9.39 ± 0.08	105.3 ± 1.7	7.14 ± 0.12 **	98.4 ± 3.5	2.3
**22**	8.99 ± 0.15	92.6 ± 1.4	7.92 ± 0.14	94.5 ± 1.7	1.0	9.40 ± 0.08	104.0 ± 1.9	7.15 ± 0.14**	97.1 ± 3.8	2.3
**24**	9.00 ± 0.15	91.8 ± 1.6	7.86 ± 0.18	93.9 ± 1.9	1.1	9.38 ± 0.07	104.6 ± 2.1	7.17 ± 0.14 **	96.2 ± 4.5	2.2
**26**	9.00 ± 0.14	90.9 ± 1.6	7.89 ± 0.14	93.1 ± 1.9	1.1	9.36 ± 0.09	104.2 ± 2.3	7.20 ± 0.13 **	94.6 ± 5.1	2.2
**28**	9.03 ± 0.13	91.1 ± 1.9	7.87 ± 0.15	93.4 ± 1.9	1.1	9.35 ± 0.09	105.3 ± 3.1	7.20 ± 0.11 *	94.0 ± 6.1	2.2
**30**	9.06 ± 0.12	91.0 ± 2.3	7.91 ± 0.10	93.1 ± 2.2	1.1	9.36 ± 0.08	105.3 ± 4.0	7.21 ± 0.11 **	93.0 ± 6.4	2.2
**32**	9.03 ± 0.13	91.6 ± 2.6	7.94 ± 0.08	93.1 ± 2.5	1.1	9.35 ± 0.06	108.0 ± 4.8	7.24 ± 0.11 **	92.1 ± 7.6	2.2
**34**	9.03 ± 0.13	93.3 ± 3.5	7.96 ± 0.07	94.3 ± 3.2	1.1	9.30 ± 0.08	111.6 ± 5.5	7.25 ± 0.13 **	90.6 ± 6.9	2.1
**36**	9.00 ± 0.15	94.7 ± 4.3	7.99 ± 0.06	94.7 ± 3.9	1.0	9.30 ± 0.07	116.2 ± 6.6	7.23 ± 0.15 **	89.7 ± 6.7	2.2
**38**	9.03 ± 0.15	95.8 ± 4.1	7.99 ± 0.05	95.1 ± 3.1	1.0	9.26 ± 0.09	121.3 ± 7.7 *	7.23 ± 0.15 **	88.5 ± 6.0	2.2
**40**	8.99 ± 0.16	98.6 ± 4.8	7.97 ± 0.05	96.2 ± 3.5	1.0	9.27 ± 0.07	125.3 ± 9.4 *	7.25 ± 0.14 **	87.6 ± 6.8	2.2
**42**	9.00 ± 0.15	102.5 ± 6.2	7.96 ± 0.03	97.6 ± 4.3	1.1	9.24 ± 0.07	134.6 ± 12.5 **	7.32 ± 0.09 *	85.9 ± 6.9	2.1
**44**	9.05 ± 0.13	105.2 ± 8.7	7.96 ± 0.04	98.0 ± 6.5	1.1	9.24 ± 0.06	145.6 ± 17.3 ****	7.31 ± 0.08 *	85.4 ± 6.8	2.2
**46**	9.19 ± 0.17	112.0 ± 10.9	7.99 ± 0.07	100.6 ± 5.2	1.3	9.23 ± 0.09	160.0 ± 23.6 ****	7.25 ± 0.09 **	84.9 ± 6.6	2.3

pEC50 ± SEM values and Emax values at both D3R mediated G protein activation and β-arrestin recruitment assays at time-points ranging from 2–46 min are reported. Bias factors, calculated as described in Section 2, are also reported. The bias factor values ≥ 2.0 are highlighted in orange. Using Sidak’s multiple comparisons tests, statistical significance are reported as ‘*’ representing significance of *p* < 0.05; ‘**’ of *p* < 0.01; ‘***’ of *p* < 0.001 and ‘****’ of *p* < 0.0001 compared to that of quinpirole.

## Data Availability

Not applicable.
